# Tooth and scale morphogenesis in shark: an alternative process to the mammalian enamel knot system

**DOI:** 10.1186/s12862-015-0557-0

**Published:** 2015-12-24

**Authors:** Mélanie Debiais-Thibaud, Roxane Chiori, Sébastien Enault, Silvan Oulion, Isabelle Germon, Camille Martinand-Mari, Didier Casane, Véronique Borday-Birraux

**Affiliations:** Institut des Sciences de l’Evolution de Montpellier, UMR5554, Université Montpellier, CNRS, IRD, EPHE, Montpellier, France; Evolution, Génomes, Comportement & Ecologie, CNRS, IRD, Univ.Paris-Sud, Université Paris-Saclay, 91198 Gif-sur-Yvette, France; Université Paris Diderot, Sorbonne Paris Cité, Paris, France

**Keywords:** Tooth, Scale, Enamel knot, Shark, *Scyliorhinus canicula*, EvoDevo

## Abstract

**Background:**

The gene regulatory network involved in tooth morphogenesis has been extremely well described in mammals and its modeling has allowed predictions of variations in regulatory pathway that may have led to evolution of tooth shapes. However, very little is known outside of mammals to understand how this regulatory framework may also account for tooth shape evolution at the level of gnathostomes. In this work, we describe expression patterns and proliferation/apoptosis assays to uncover homologous regulatory pathways in the catshark *Scyliorhinus canicula*.

**Results:**

Because of their similar structural and developmental features, gene expression patterns were described over the four developmental stages of both tooth and scale buds in the catshark. These gene expression patterns differ from mouse tooth development, and discrepancies are also observed between tooth and scale development within the catshark. However, a similar nested expression of Shh and Fgf suggests similar signaling involved in morphogenesis of all structures, although apoptosis assays do not support a strictly equivalent enamel knot system in sharks. Similarities in the topology of gene expression pattern, including Bmp signaling pathway, suggest that mouse molar development is more similar to scale bud development in the catshark.

**Conclusions:**

These results support the fact that no enamel knot, as described in mammalian teeth, can be described in the morphogenesis of shark teeth or scales. However, homologous signaling pathways are involved in growth and morphogenesis with variations in their respective expression patterns. We speculate that variations in this topology of expression are also a substrate for tooth shape evolution, notably in regulating the growth axis and symmetry of the developing structure.

**Electronic supplementary material:**

The online version of this article (doi:10.1186/s12862-015-0557-0) contains supplementary material, which is available to authorized users.

## Background

### Tooth morphogenesis and evolution in mammals

Teeth have been a constant object of study in developmental biology because of their histological simplicity and autonomous development. As the most highly mineralized piece of vertebrate anatomy, teeth also represent the most commonly fossilized object for vertebrate paleontologists. This link between evolutionary and developmental biology is currently very productive through studies of the developmental processes involved in tooth shape variation in evolutionary times [1, 2]. In mouse, numerous developmental genetic studies have deciphered how tooth initiation, morphogenesis and differentiation are controlled through reciprocal inductive interactions between both epithelial and mesenchymal compartments. These interactions involve the synthesis of signaling molecules and transcription factors with regionalized and temporally restricted expression patterns [3].

At the histological level, tooth development is usually characterized by four subsequent stages in mouse and other vertebrate models [4–6]. The first step is early morphogenesis (EM) when a focal surface of a specialized epithelium (the dental lamina or odontogenic band) thickens and signals towards the mesenchymal compartment. The mesenchymal cells, partly derived from neural crest cells, condensate under this epithelial placode. This step necessitates the expression of genes involved in the Bone Morphogenetic Protein (Bmp) signaling pathway (in mammals, *Bmp2* and *Bmp4*) which activates cell differentiation, but also the Hedgehog and Fibroblast Growth Factor (Fgf) signaling pathways which induce proliferation and counteract the Bmp pathway [7–10]. The expression of transcription factors such as Pitx, Msx and Dlx is associated with this first step of tooth development through the specification of the dental epithelium and mesenchyme [11–16]. Epithelial at first, these signals induce their own expression and expression of specific genes in mesenchymal cells that themselves induce the morphogenesis of the tooth bud.

Cell proliferation drives morphogenesis of the growing bud during the late morphogenesis step (LM, also named cap-stage in the mouse) and is characterized in mammals by the presence of a transient signaling center in the inner dental epithelium, named the primary enamel knot [17], which induces regionalized proliferation of both epithelial and mesenchymal cells leading to the first acquisition of the tooth bud shape. This step is also regulated by the Bmp, Shh and Fgf signaling pathways, with very localized expression of several genes in the enamel knot [9, 10, 17–19]. In mammalian molars, secondary enamel knots further regulate the folding and growth of the epithelial cell sheet [17], modeling the shape of the surface between the epithelial and the mesenchymal cells (stage named Early Differentiation, ED). The cell differentiation stage (named Late Differentiation, LD) starts at the cusp tip while morphogenesis is still on-going. The first visible signs are given by the appearance of polarized ameloblasts (that synthesize an extracellular matrix which will eventually be mineralized and give rise to the enamel layer in mammals) from the epithelial compartment while cells from the mesenchymal compartment differentiate into odontoblasts (which synthesize an extracellular matrix that will give rise to the mineralized dentin).

The position of the enamel knots are therefore supposed to regulate precisely the shape of the epithelium-mesenchyme boundary through reiteration of pro- and anti-proliferation signals in mammals [10, 20, 21]. The shape of the epithelium-mesenchyme boundary determines the final shape of the enamel surface. These signaling actions have been reduced to a simple activator-inhibitor feedback loop in computational modeling studies [22–24]. In this model, two diffusible epithelial signals represent anti-proliferation (Wnt, Bmp) and pro-proliferation (Fgf, Shh) forces acting on local epithelial and mesenchymal cells. The presence of these signals are sufficient to obtain observed tooth shapes and to account for variation of cusp shape and number observed in mammalian teeth [22, 24]. Among all acting parameters of tooth morphogenesis, epithelial growth and its regulation by the enamel knot source of diffusible signals has a major effect. This model represents a strong explicative tool to describe modifications in regulatory cascades that may account for the evolution of tooth structures [1, 24, 25].

Two transcription factor families were shown to be involved in the regulation of the Bmp signaling pathway during tooth development, the Pitx and the Msx families. *Pitx2*^*−/−*^ mutant mice display tooth development arrest at an early stage [15]. However *Pitx1*^*−/−*^ mutant mice do not show this loss of tooth development, although *Pitx1* gene is expressed early in the dental epithelium. The Pitx proteins bind and regulate *Bmp4* expression in tooth epithelium [13]. The description of this control region let the authors suggest that Pitx2 had a negative regulatory activity on *Bmp4* while Pitx1 could be a positive regulator of this gene in the dental epithelium. This enhancer is active in the dental epithelium but transgene expression is excluded from the enamel knot, which contrasts with the endogenous *Bmp4* expression pattern. This result suggests that an additional regulatory sequence of *Bmp4* is involved in the activation of transcription in the enamel knot expression [13]. In the same study, the binding of Msx transcription factors was detected on the same regulatory sequence. In particular, Msx1 has been described in the regulation of epithelial-mesenchymal signaling through Bmp4 expression. *Msx1*^*−/−*^ mutant mice display tooth development arrest and a loss of *Bmp4* mesenchymal expression [12]. Classically, an enamel knot is defined as non-proliferative epithelial cells which co-express Bmp, Fgf and Shh genes, and finally undergo apoptosis.

### Tooth morphogenesis outside of mammals

The signaling pathways involved in tooth development in mammals have been shown to be conserved outside of mammals, notably in diapsids [26, 27]. However, no evidence of the presence of an enamel knot in teleost fish has been proposed even though all classical signaling pathways described in the enamel knot are expressed in the tooth epithelium, e.g. Shh and Bmp [28, 29] or *Dlx* genes [6, 30].

Functional studies, mostly in zebrafish, have shown th at the Shh signaling pathway is active during tooth development [31]. Its involvement could be tested in tooth initiation and mineralization but not in tooth morphogenesis, because teeth are unicuspid in zebrafish. However, teeth of other adult teleost fish may display variations in the number and shape of cusps [32] and over-expression of the Fgf or down-regulation of the Bmp pathway led to the development of multicuspid teeth in larval zebrafish and Mexican tetra [33]. These results suggest that although an enamel knot is not morphologically observable in teleosts, the regulation of tooth shape through cusp development may be shared between mammals and teleosts.

Teleost fish were chosen as an out-group to mammals and other tetrapods because a series of new model species for evolutionary developmental biology have emerged in this group, such as zebrafish, Mexican tetra and cichlids. Much less research has explored the genetic regulation of tooth morphogenesis outside of bony vertebrates, i.e. in cartilaginous fish. The extant cartilaginous fish group includes (i) holocephalans (tooth plates made of fused teeth, no single teeth), and (ii) neoselacians that group together sharks, rays and skates (dentition made of a large number of single teeth that are permanently renewed, great variation in tooth shape) [34]. Among cartilaginous fish, the small spotted catshark *Scyliorhinus canicula* has become a reference species in evolutionary developmental biology [35]. Tooth development in the catshark has been described at the histological and molecular level with emphasis on the developmental similarities between teeth on the jaw and scales on the skin [4, 36, 37]. However, the exploration of putatively conserved signaling pathways found in the mammalian enamel knot has not been proposed yet. Working with catshark embryos allows the access to successive tooth buds on one individual, with embryonic teeth already displaying cusps, and with morphologically identified developmental stages for teeth and scales [4]. In addition, tooth and scale buds display very similar expression patterns of regulatory genes at their initiation and morphogenesis stages, while the final shape of these structures is very different [4]. This situation offers an excellent internal control for the identification of the signaling pathways involved in tooth shape acquisition in chondrichthyans, through the comparison between a structure with cusps (teeth) and a structure without cusps (scales) within the same organism.

In this study, we collected a series of data on tooth and scale bud development in the catshark and compared them to the mouse molar enamel knot system: we describe gene expression patterns for a selected series of enamel knot markers from the Bmp, Fgf, Shh, Msx and Pitx signaling pathways, as well as data on proliferation and apoptosis dynamics in the epithelium and mesenchyme of these structures. Our results do not support the presence of a strict equivalent to an enamel knot in the small-spotted catshark tooth buds. On the other hand, they open new questions about the gene regulatory cascades involved in the symmetry of tooth development.

## Methods

### Tooth and scale morphology

Heads of dead adult catsharks (*Scyliorhinus canicula*) were obtained as leftovers on a fishmarket in the west of France, Roscoff (no field work permission needed). Jaws were prepared by removal of most of the skin and flesh, then air-dried. Single teeth were sampled on jaws, and coated with platine prior to SEM observation, which was performed at the Institut Européen des Membranes, Montpellier (CNRS, UM, ENSCM) with a Hitachi S-4800 using an acceleration voltage of 10 Kv. Embryonic jaws and tail buds were sampled on fixed embryos (7,5 and 4,8 cm long embryos, see next section for embryo collection). They were rinsed in several phosphate buffered saline solution (PBS) 1X bathes, and a KOH 0,5X bath and then stained in 0,001 % alizarin red in a 0,5X KOH solution, overnight. Stained specimens were transferred in graded series of KOH 0,5X/glycerol and then stored and imaged in 100 % glycerol.

### Embryos collection and staging

Catshark embryos were obtained from the Station de biologie de Roscoff, France (Service d’Expédition de Modèles Biologiques - CNRS-UPMC/FR2424). Collection and handling of animals was carried out in full compliance of institutional (local committee #59 of the Ministère de l’Enseignement Supérieur et de la Recherche, France), national and international guidelines (European Communities Council Directive of 22 September 2010 (2010/63/UE)) and did not require approval by an ethics committee. All embryos were maintained at 17 °C in sea water at the CNRS animal husbandry facility in Gif-sur-Yvette (facility reference C 91 272 105) until they reach a given developmental stage, defined by their total length. They were euthanized with MS222, dissected and then fixed 48 h *for* in situ hybridization or overnight for immuno-detection, at 4 °C in 4 % paraformaldehyde (PFA) in PBS. Embryos were then dehydrated in methanol and stored at −20 °C.

To observe gene expression at the four characteristic developmental stages of tooth and scale, whole mount in situ hybridizations were performed respectively on dissected lower jaws of stage 32 embryos (body length ranging from 3,8 to 5,5 cm [38, 39]) and dissected tails of stage 29 embryos (body length ranging from 2,5 to 3 cm).

### cDNA sequences

We have identified eight genes orthologous to mammalian odontogenesis developmental genes in a cDNA library from *Scyliorhinus canicula* [40] through a BLAST analysis with mouse sequences of the Bmp, Fgf, Msx and Pitx gene families. We identified sequences belonging to all four gene families, and they were each assigned to one orthology group through phylogenetic reconstruction of gene trees after sequence alignment to sequences identified in other jawed vertebrate genomes (see Additional file 1 for phylogenies). These sequences are identified as *ScBmp4* (partial in NCBI [Genbank: EF174300.1]), *ScFgf8* (partial in NCBI [Genbank: DQ647321.1]), *ScMsx1, ScMsx2*, *ScMsx3*, *ScPitx1* [Genbank: KJ190312.1], *ScPitx2* [Genbank: KJ190313.1] and *ScPitx3* (partial in NCBI [Genbank: KJ190314.1]). The *ScShh* sequence previously published [37] was subcloned and used as an additional marker in this study. We amplified selected sequences of these cDNAs and published the new partial sequence in NCBI: ScBmp4 [Genbank: KT261786]; ScFgf8 [Genbank: KT261787]; ScMsx1 [Genbank: KT261788]; ScMsx2 [Genbank: KT261789]; ScMsx3 [Genbank: KT261790]; ScPitx3 [Genbank: KT261791]. ScPitx1 and ScPitx2 sequences were amplified from the primers Sc-Pitx1-F (ACAGGCTTTCATATGTTCGG), ScPitx1-R (TGCTGCCGCCTCCGTGTCCG), ScPitx2-F (GGGATCCTTATCTGCAGTTA) and ScPitx2-R (CTCCCGTGTCAGGGCTCGAG) and their sequence is included in KJ190312.1 and KJ190313.1 respectively.

### Catshark probes and in situ hybridization

Antisense RNA digoxigenin-UTP probes were transcribed using SP6 or T7 RNA polymerases (Roche), according to the orientation of the insert in the plasmid. In situ hybridizations were performed on dissected catshark lower jaws and dissected tails as previously described [41] with proteinase K treatments (10 μg/ml) as in [4]. The color detection step was performed using the NBT-BCIP reaction (Roche). Samples were post-fixed in 4 % PFA after whole mount in situ hybridization, then cleared and stored in glycerol at 4 °C until photographed.

### Histological sectioning

Whole-mount hybridized samples were put through several baths of absolute ethanol, then in butanol and finally embedded in paraplast for 10 μm cross-sections. For histological analysis, hybridized jaws were then cut longitudinal and hybridized tails were cut transversal. Negative whole-mount detections were also verified after histological sections.

### Proliferating cell nuclear antigen (PCNA) proliferation assays

Dissected tails (embryos from 2.7 to 3.2 cm long) or jaws (embryos between 4.9 and 6,6 cm long) were demineralized for 1–2 h respectively in MORSE solution (sodium citrate 10 % and formic acid 20 %) at room temperature prior to dehydratation, embedded in paraplast and cut to 10 μm thickness. PCNA immuno-staining was performed using a primary anti-PCNA dilution at 1:500 (P8825, Sigma). For epitope retrieval, sections underwent microwave-induced heat treatment in Tris EDTA buffer at pH9/tween 20 0.05 % (40 s at 600 W until boiling and then 20 min at 120 W). Cell nuclei were counterstained with Hoechst (Sigma).

### Apoptosis detection

Dissected whole-mount jaw (4,8 cm long embryo) and tail (3 cm long embryo) were fixed for 30 min with 4 % PFA and then in ethanol 100 % at −20 °C. After rehydration to PBS-tween 0.1 %, they were permeabilized for 30 min in a Proteinase K solution (10 μg/mL) and then transferred in 1 % triton X-100 overnight, both in PBS solution at room temperature. TUNEL staining (In situ cell death detection kit, TRITC, Roche) was used according to the manufacturer’s instructions. Indirect immunofluorescence was performed with 1 % Phalloidin-FITC (binds polymeric F actin, Sigma-Aldrich) in PBS solution, 0.01 % triton. Nucleus DNA was stained with 0.5 μg/ml DAPI (4′,6-diamidino-2-phenylindole, Life technologies). Specimens were analyzed with a Leica TCS-SPE laser confocal microscope (Montpellier RIO Imaging platform, France).

## Results

### Variations in tooth and scale morphology in the catshark

**Fig. 1:**
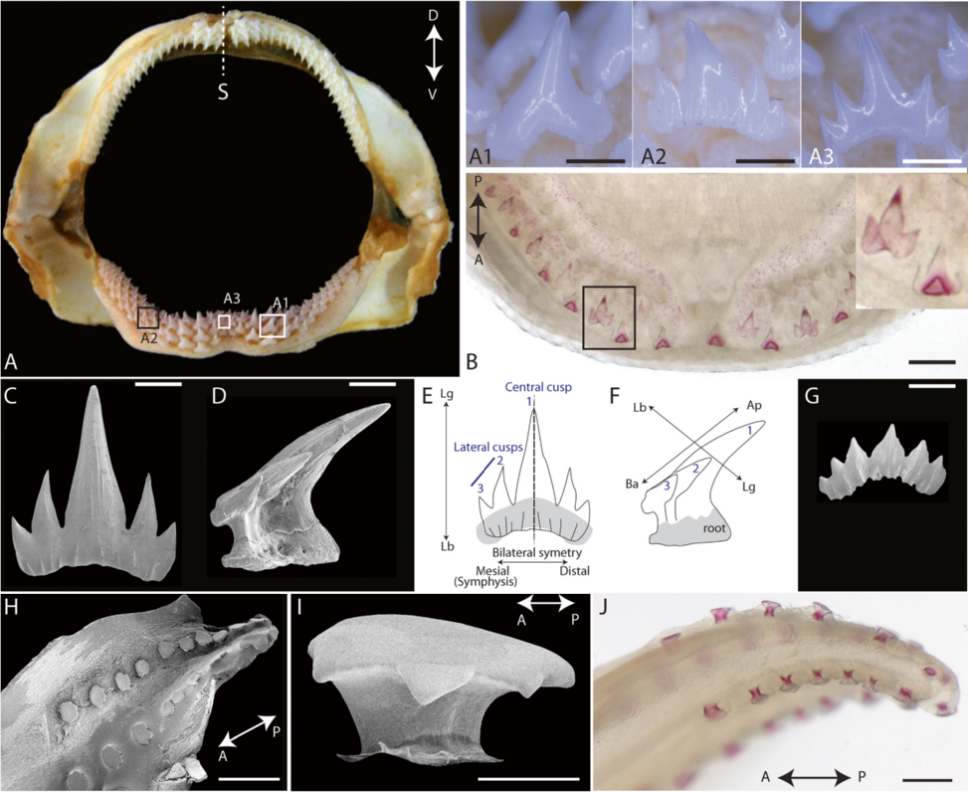
External morphology of adult and developing teeth and scales in *Scyliorhinus canicula*. **a** adult male jaw, frontal view with insets on teeth from the lower jaw (A1, tooth from the lateral side, A2, para-symphyseal tooth, A3, symphyseal tooth). **b** dorsal view of an alizarin red stained lower jaw of a 7.5 cm long embryo with inset on the tricuspid mineralized first generation teeth. **c** SEM, labial view of a bilaterally symetric tooth with five cusps from an adult female, **d** SEM, lateral view of a similar tooth. **e** and **f**: schematics of teeth in **c** and **d** with crown orientation: ap: apical; ba: basal; lb: labial; lg, lingual. **g**: SEM, tooth of an adult female with altered symmetry and small central cusp. **h**: SEM, lateral view of the tip of the tail of a 4.8 cm long embryo showing the caudal scales. **i**: SEM, lateral view of one erupted caudal scale. **j**: ventral view of the tail of a 5 cm long embryo after staining with alizarin red showing developing caudal scales. Scale bars: A1, A3, B, H: 500 μm; A2: 700 μm; C, D, G, J: 250 μm; I: 50 μm

The observation of adult jaws of the catshark *Scyliorhinus canicula* showed a regular organization of their dentition: teeth were organized in families with the older tooth localized in a rostral (labial) position while more recently developed teeth (successive replacement teeth) were observed in a more caudal (lingual) position (Fig. 1a, and see supplementary material in [37]).

Individual teeth were bent on a labial to lingual direction and showed a long central cusp with various numbers of lateral smaller cups (Fig. 1a1-3, c-g). On adult male jaws, the para-symphyseal teeth displayed two lateral cusps (Fig. 1a1) with occurrence of additional cusps on teeth farther from the symphysis or located right at the symphysis (Fig. 1a2 and a3). Sexual dimorphism leads to tooth shape variation: female teeth usually display more cusps than male teeth (Fig. 1c, g, and [42]). Overall, symphyseal and para-symphyseal teeth displayed a bilateral symmetry with a long axis (labial-lingual axis) following the anterior-posterior axis of the body and a perpendicular axis (apical-basal) following the dorsal-ventral axis of the body (Fig. 1c, e). Note that this bilateral symmetry was often modified when a tooth was taken in a more lateral position in reference to the symphysis, in association with more cusps observed (compare Fig. 1c and g). During catshark development, the first externally visible tooth bud generally appeared in 4 cm long embryos on each hemi lower jaw and the first mineralized tooth could be detected in embryos reaching 6 cm long [4]. In a 7.5 cm long embryo, five to six mineralized teeth were present on each hemi jaw: each tooth showed three cusps and was bilaterally symmetrical (see Fig. 1b). No sexual dimorphism in tooth shape has been detected in juveniles of *Scyliorhinus canicula* [43].

The first developing dermal scales (caudal primary scales) were organized into four rows at the tip of the tail, two dorsal and two ventral, displaying respectively ten and eight scales on average (see Fig. 1h, and [4, 44]). The top of caudal primary scales was a flat surface with an irregular outline supported by a root: the overall symmetry appeared radial even though a slight lengthening of the flat surface was visible along the antero-posterior axis of the body (Fig. 1i). During catshark development, the first caudal primary scales were observable in 2.5 cm long embryos as bilateral buds and then progressively developed in a posterior to anterior wave [4]. In a 5 cm long embryos, the full set of caudal primary scales (from 8 to 12 in each row) was mineralized (Fig. 1j and [4]).

### Early tooth development and bud growth

**Fig. 2:**
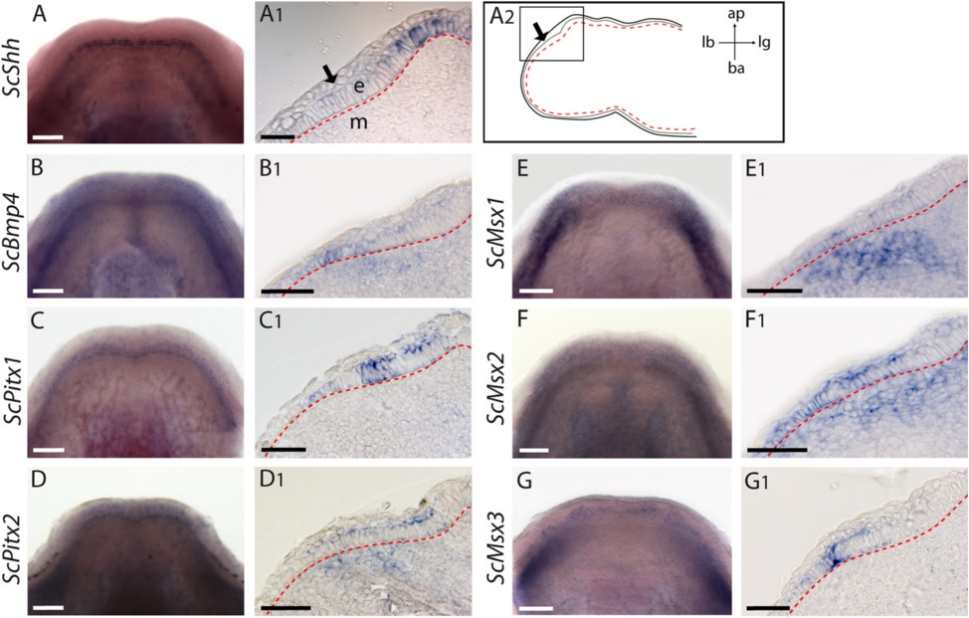
Gene expression patterns prior to tooth initiation in the catshark *Scyliorhinus canicula.* Expression is seen on whole mount lower jaws (**a–g**) and longitudinal sections (**a1–g1**) showing tissue specific expression in the odontogenic band (*black arrow*) except for Sc-*Shh*. A schematic of the whole section surface, with orientation, is given in **a2**. The square indicates the region magnified in **a1-g1**. Gene names are indicated of the left side of the panel. The basal membrane is located with a dotted red line. ap: apical, ba: basal, e: epithelium of the odontogenic band, lb: labial, lg: lingual, m: mesenchyme. Scale bars: (**a–g**) 200 μm, (**a1-g1**) 50 μm

Before any sign of tooth bud development (stage late 31; body length from 3,8 to 4,2 cm; [38]), *ScBmp4*, *ScMsx1*, *ScMsx2*, *ScMsx3*, *ScPitx1* and *ScPitx2* are continuously expressed along the odontogenic band region, the area where teeth will develop (Fig. 2). Note that no corresponding stage could be defined in scale bud development. Histological sections showed that *ScBmp4*, *ScMsx1*, *ScMsx2* and *ScPitx2* transcripts are localized in a broad area in both the epithelium and mesenchyme of the odontogenic band region, while *ScPitx1* and *ScMsx3* transcripts are only restricted to a small area in the odontogenic epithelium (Fig. 2). As previously described [37], we observed an epithelial *ScShh* expression in a more lingual position than the other genes, but no transcripts could be detected in the area where teeth develop in the odontogenic band (Fig. 2).

**Fig. 3:**
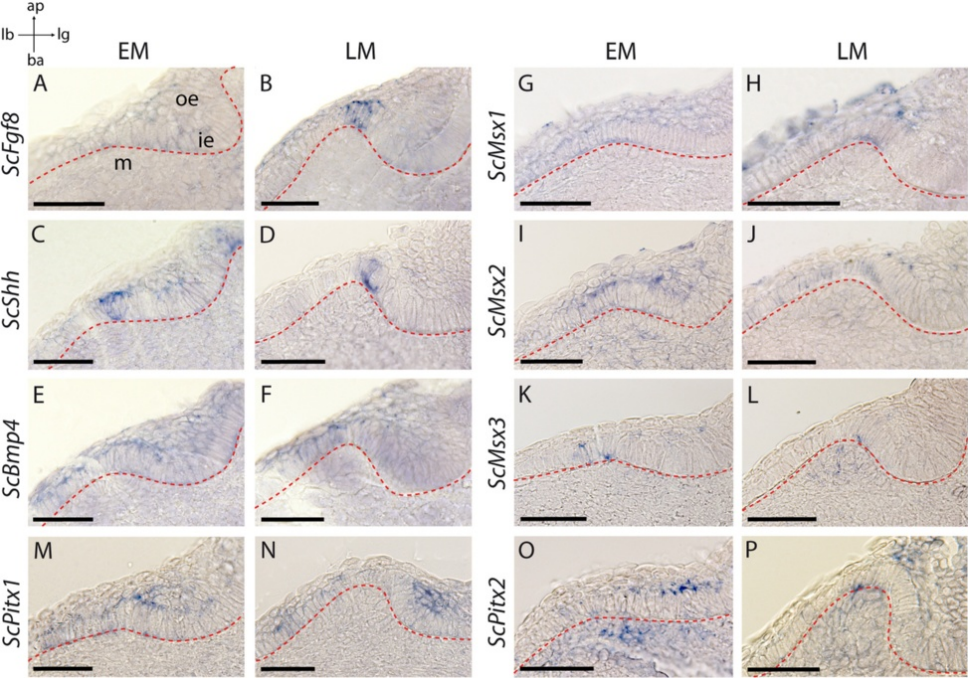
Gene expression patterns during early tooth development in the catshark *Scyliorhinus canicula*. Longitudinal sections showing tissue specific expression in tooth buds at stage EM (**a, c, e, g, i, k, m, o**) and LM (**b, d, f, h, j, l, n, p**). Gene names are indicated of the left side of each panel, the basal membrane is located with a dotted red line. ap: apical, ba: basal, ie: inner epithelium, lb: labial, lg: lingual, m: mesenchyme, oe: outer epithelium. Scale bars: 50 μm

Early and late morphogenesis stages of developing teeth (EM and LM) could be observed on the lower jaw of embryos ranging from 4.2 to 4.7 cm. All investigated genes, except *ScPitx3*, were expressed during tooth morphogenesis (Fig. 3). On whole mount in situ hybridizations, *ScFgf8* and *ScShh* had spatially restricted expression patterns in the first developing teeth (observable as discrete dots, data not shown). Sections of these whole-mount in situ hybridizations showed that *ScShh* was expressed during EM and LM, while transcription of *ScFgf8* started during LM (Fig. 3a–d). In both cases, transcripts were restricted to few inner dental epithelial cells at the tip of the developing tooth bud. In situ hybridizations against *ScBmp4* showed a fainter signal located in the outer dental epithelium of tooth buds at EM and LM, with faint expression in the inner dental epithelium at stage LM (Fig. 3e–f). This expression is located asymmetrically in the labial part of the tooth.

*ScMsx1, ScMsx2 and ScMsx3* genes were expressed in broader round territories, each of them corresponding to a developing tooth bud (data not shown). Histological sections (see Fig. 3g–l) revealed that all these genes were still expressed during morphogenesis (EM and LM) in the inner dental epithelium although *ScMsx3* displayed a very restricted zone of expression (Fig. 3k–l). *ScMsx1* and *ScMsx2* were also expressed in the outer dental epithelium (Fig. 3g–j). *ScMsx3* displayed a localized zone of expression at the tip of the tooth bud (Fig. 3k-l), similar to the *ScShh* zones of expression. *ScMsx2* was expressed in the whole jaw mesenchyme at stage EM (Fig. 3i) and its expression appeared fainter at the LM stage while *ScMsx3* started to be expressed in the dental mesenchyme only at the LM stage (Fig. 3l).

On whole-mount jaws, *ScPitx1* was more strongly expressed in a continuous band surrounding the tooth buds, with poorly detectable expression in the developing tooth buds themselves (data not shown). Histological sections showed expression of *ScPitx1* only in the epithelial compartment, mostly in the outer dental epithelium at stages EM and LM but also in the labial part of the inner dental epithelium (Fig. 3m–n). *ScPitx2* was also expressed in the outer dental epithelium of EM and LM tooth buds, as well as in the mesenchyme of tooth buds (Fig. 3o–p).

### Expression during the acquisition of tooth morphology

**Fig. 4:**
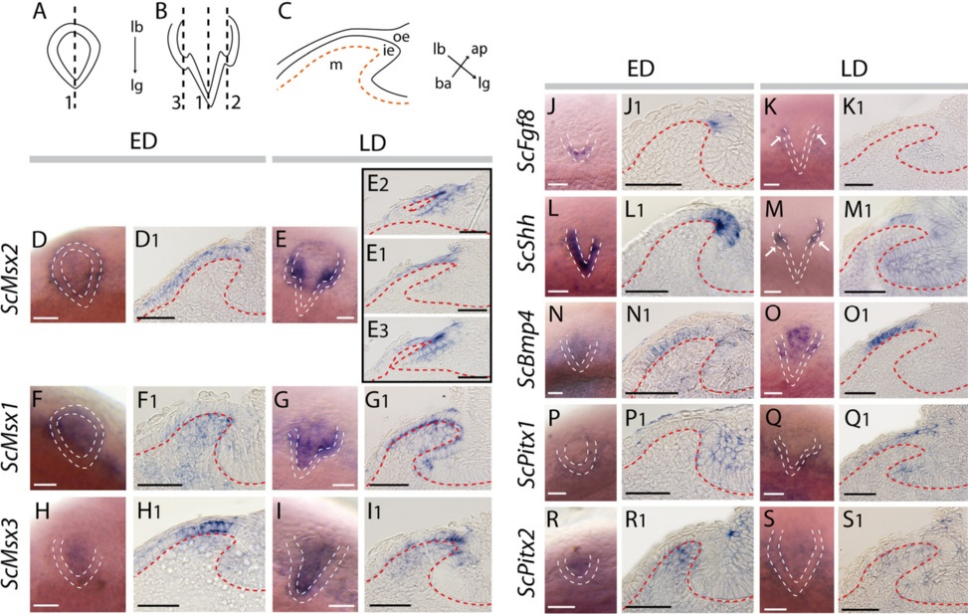
Gene expression patterns during late tooth development in the catshark *Scyliorhinus canicula*. **a** and **b** schematics of tooth bud at respectively ED and LD stage, dorsal view, labial side to the top. **c** schematic of histological section following section plane 1. Whole-mount dorsal views of tooth buds at ED and LD stages (**d–s**) and longitudinal sections following section plane 1 showing tissue specific expression (**d1–s1**). **e2** and **e3** longitudinal sections following section planes 2 and 3. Gene names are indicated of the left side of the panel, same legends as Fig. 3. Scale bars: 50 μm

Early and late differentiation stages (ED and LD respectively) of developing teeth could be observed in embryos ranging between 4,8 and 5,5 cm. The developing central cusp could be observed in teeth at ED stage (see Fig. 4a) showing that tooth morphogenesis is still on-going. Developing lateral cusps were observed at the LD stage (see Fig. 4b).

All the investigated genes, except *ScPitx3*, were expressed during the ED and LD stages (Fig. 4). On whole mount jaws, during the development of the central cusp, restricted expressions could be detected with *ScFgf8* (marking the tip of the developing bud, Fig. 4j), *ScShh* (V shape staining the outline of each developing tooth, Fig. 4l), and *ScMsx3* (focal expression in the tooth bud, Fig. 4h). *ScMsx1*, *ScMsx2*, *ScBmp4* and *ScPitx2* expressions were restricted to spots covering each developing tooth bud during the ED stage (Fig. 4d, f, n and r). *ScPitx1* expression seemed stronger in the zone surrounding the tooth buds (Fig. 4p). At LD stage, when developing lateral cusps appeared, most of genes were no more expressed at the tip of the central cup but expression was maintained in the two forming lateral cusps (Fig. 4).

Histological sections showed that *ScFgf8*, *ScShh* and *ScBmp4* were only expressed in the inner dental epithelium during the ED stage, with *ScShh* and *ScFgf8* transcripts restricted in few cells at the tip of the developing central cusp and *ScShh* being expressed in a broader area than *ScFgf8* (Fig. 4j1, l1 and n1). Expression of these two genes was not observed at the LD stage in the central cusp (Fig. 4k1 and m1) but could be detected in the lateral cusps (Fig. 4k and m, arrows). *ScBmp4* transcripts were found in the labial part of the inner dental epithelium excluding the tooth basis and the tooth tip at stage ED and LD (Fig. 4n1 and o1).

During the ED stage, *ScMsx2* transcripts were restricted to the labial side of the inner dental epithelium (Fig. 4d1). *ScMsx1* and *ScMsx3* were expressed in both the inner dental epithelium and the dental mesenchyme (Fig. 4f1 and h1): in the epithelial layer, *ScMsx3* transcripts were localized on the labial side of the developing tooth whereas *ScMsx1* transcripts were found in the whole tooth. At the LD stage, epithelial expression of these three genes was no more observed at the tip of the central cusp while it could be observed in both lateral cusps (Fig. 4e1-e3, g1, i1).

At stage ED, *ScPitx1* transcripts were found in the inner and outer dental epithelia on the lingual side of the developing tooth (Fig. 4p1) whereas *ScPitx2* transcripts were found in the dental mesenchyme and in the outer dental epithelium facing the tip of the tooth bud (Fig. 4r1). Later, at stage LD, *ScPitx1* and *ScPitx2* transcripts were detected in the outer epithelium of the labial and lingual sides of the developing tooth (Fig. 4s1, q1). At this same stage, faint expression of *ScPitx1* could also be detected in the inner epithelium, on the labial side of the tooth (Fig. 4q1) and *ScPitx2* expression was barely detectable in the dental mesenchyme (Fig. 4s1).

### Dynamics of gene expression patterns during scale development

**Fig. 5:**
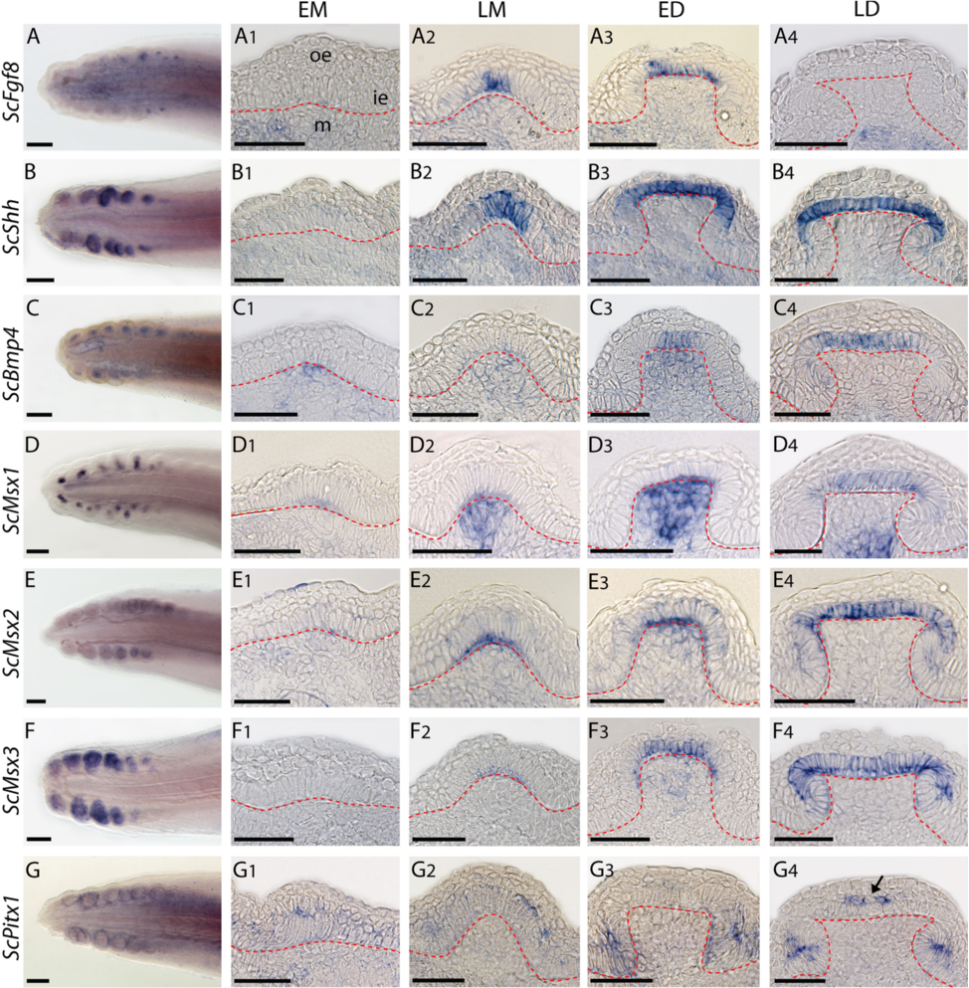
Gene expression patterns during dermal scale development in the catshark *Scyliorhinus canicula*. Whole-mount hybridized tails of about 3 cm long embryos (**a–g**) and transverse sections showing tissue specific expression in scale buds at stage EM (**a1–g1**), LM (**a2–g2**), ED (**a3–g3**) and LD (**a4–g4**). Gene names are indicated of the left side of each panel, same legends as Figs. 3 and 4. Scale bars: (**a–g**) 200 μm, (**a1–g4**) 50 μm

Seven out of the nine investigated genes were expressed during the development of the caudal primary scales: *ScShh*, *ScBmp4*, *ScFgf8*, *ScMsx1, ScMsx2*, *ScMsx3*, and *ScPitx1* (Fig. 5a–g) while *ScPitx2* and *ScPitx3* transcripts could not be detected (data not shown).

*ScShh* and *ScFgf8* transcripts displayed epithelial-specific expression patterns: no expression was detected at the stage of Early Morphogenesis (EM), when a focal thickening of the epithelial is visible, with condensation of mesenchymal cells on its basal pole (Fig. 5a1, b1). Their transcription started in the bud epithelium at the Late Morphogenesis stage (LM), when interactions between the epithelium and mesenchyme allow the growth of an actual bud (Fig. 5a2, b2). We considered the start of Early Differentiation (ED) when constriction at the basal zone of the scale bud could be observed: transcripts of *ScShh* and *ScFgf8* were still restricted in the bud epithelium at that stage, with *ScFgf8* transcripts more restricted to the center of the apical zone of the scale bud (Fig. 5a3, b3). Later expression of *ScShh* was detected at the Late Differentiation (LD) stage on the whole apical surface, when the scale bud epithelium folds and clearly defines the apical and root zone of the future scale (Fig. 5b4). This stage of differentiation is also the time when epithelial cells of the bud differentiate into secreting ameloblasts [4]. No expression of *ScFgf8* could be detected at this stage (Fig. 5a4).

*ScBmp4* displayed an apically restricted zone of expression in the bud epithelium at the ED and LD stages (Fig. 5c3, c4). It differed significantly with the previous expression patterns in that expression in the mesenchymal cells of the scale bud could be detected from the EM to the ED stage (Fig. 5c1-3).

*Msx* genes had contrasted expression patterns. Transcripts of *ScMsx1* were located mostly in the mesenchymal compartment (stage LM to LD) with transitory expression in the center of apical epithelial cells of the scale bud at stage LD (Fig. 5d1-4), similar to *ScBmp4. ScMsx2* transcripts were also detected in the whole apical surface of epithelium (stage LM to LD) and transitorily in the mesenchyme at stage ED (Fig. 5e1-4). Finally, transcripts of *ScMsx3* had a mostly epithelial expression pattern very similar to the *ScMsx2* and *ScShh* expression patterns (Fig. 5f1-4). Faint expression was detected first at stage LM (Fig. 5f2), then strongly at stage ED in the apical epithelial cells of the bud (Fig. 5f3). Transient expression in the mesenchyme could also be detected at this stage.

*ScPitx2* and *ScPitx3* transcripts could not be detected during scale bud development, but *ScPitx1* transcripts were located only in the epithelial compartment, and mostly outside of the scale bud (Fig. 5g1-4). We called this zone the outer epithelium after the mouse outer dental epithelial and hypothesized to be comparable to the pharyngeal epithelium surrounding teeth in the zebrafish [6]. Transcripts of *ScPitx1* were detected at low levels in the scale bud epithelium and outer epithelium at stages EM and LM (Fig. 5g1-2) and then restricted to the basal folding zones of the scale bud at ED (Fig. 5g3) and in the outer epithelium in contact with the center of the apical bud epithelium (Fig. 5g4, arrow).

### Cell proliferation and apoptosis during tooth and scale development

**Fig. 6:**
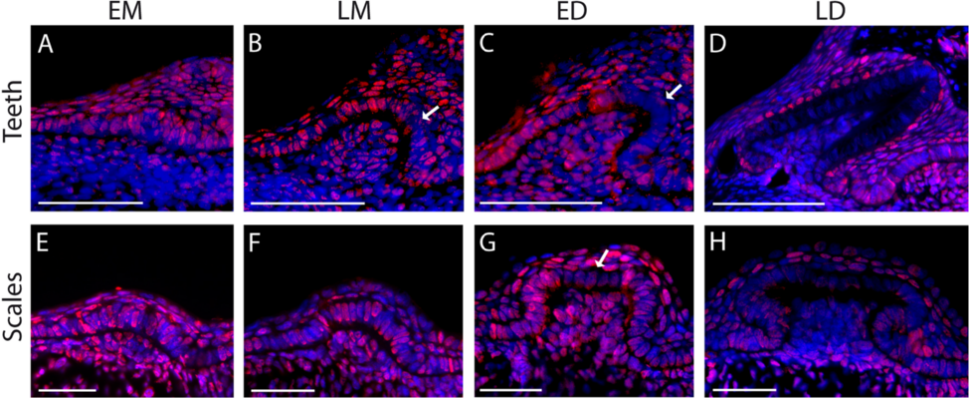
Cell proliferation pattern during tooth bud (**a–d**) and scale bud (**e–h**) development in the catshark *Scyliorhinus canicula*. **a–d** Histological sections of dissected jaws. **e–h** Histological sections of dissected tails. PCNA staining appears red and counter-staining of the nuclei is *blue*. Developmental stages are indicated on the figure. Scale bars: 50 μm

As another way to detect the specific region of a putative enamel knot in the catshark, we tested proliferation and apoptosis in tooth and scale buds. The localization of proliferation areas was investigated by immunostaining with an antibody against the proliferating cell nuclear antigen (PCNA, Fig. 6). At the beginning of tooth morphogenesis, odontogenic and non-odontogenic epithelium and the underlying condensing mesenchyme were highly proliferative (Fig. 6a). During late morphogenesis and early differentiation, a non-proliferative area appeared at the tip of the tooth epithelium (Fig. 6b, c, arrow). In later stages of teeth development, this area extended as the ameloblast differentiation progressed and proliferation decreased in teeth mesenchyme (Fig. 6d). The proliferation dynamic in scale buds seemed homogenous in both the epithelium and mesenchyme, with stronger proliferation during late morphogenesis and early differentiation except in the apical zone of the epithelium at ED (Fig. 6f, g, arrow). During late differentiation, proliferation was stronger in the inner epithelium of the root than at the top of the scale bud and very low in the mesenchyme (Fig. 6h).

**Fig. 7:**
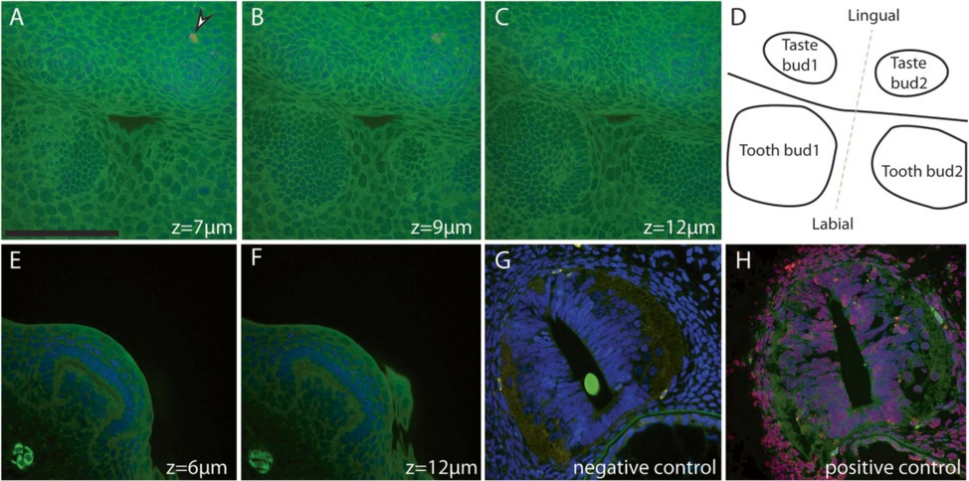
Apoptosis detection in developing tooth and scale buds in the catshark *Scyliorhinus canicula*. Apoptotic cells (TUNEL, *red*), actin (phalloidin, *green*) and DNA (DAPI, *blue*) were localized by triple labelling and confocal microscopy imaging. Dorsal views of successive z-planes of a whole-mount lower jaw, merged for all three canals (**a**, buccal surface with taste buds; **b**, dental epithelium layer of tooth bud 1, late LM; **c**, dental epithelium layer of tooth bud 2, late LM; **d**: schematic of the dorsal view). Lateral views of successive planes in a caudal scale, merged for all three canals, early ED (**e**, scale bud side, z = 6 μm; **f**, scale bud center, z = 12 μm). Sections through the neural tube merged for all three canals, **g** negative control without the terminal deoxynucleotidyl transferase (TdT) enzyme, no apoptosis is detected; **h** positive control after DNAse I digestion, all nuclei are positively stained with both the DAPI and the TUNEL, resulting in purple fake color. White arrowhead: TUNEL positive staining in the buccal epithelium. See Additional files 2 and 3 for all z-planes. Scale bar: 150 μm

Apoptosis detection on developing jaws and tails led to no staining in either tooth buds or scale buds which could be staged at LM or ED while other sites of apoptosis could be detected in the surface ectoderm of the mouth (see the Fig. 7 and animations on Additional files 2 and 3).

## Discussion

In order to compare our results to published data on mouse molar development, we present two summary tables for gene expression in the mesenchyme (Table 1) and epithelium (Table 2). We compared the stages of mouse molar growth to shark tooth and scale buds through the following time points: (i) EM includes the initiation and bud stage; (ii) LM corresponds to the cap stage during which the primary enamel knot is active in mouse; (iii) ED is comparable to the beginning of the bell stage, when secondary enamel knots form and the definitive shape of the bud is acquired; (iv) LD includes the late part of the bell stage, starting when ameloblasts are morphologically differentiated.

**Table 1:** Summary of mesenchymal gene expression patterns

Stage	EM			LM			ED			LD		
	Scale	Tooth	Molar	Scale	Tooth	Molar	Scale	Tooth	Molar	Scale	Tooth	Molar
*Bmp4*	+	-	+ [46]	+	-	+ [46]	+	-	+ [46]	-	-	+ [46]
*Msx1*	-	-	+ [61]	+	-	+ [61]	+	+	+ [61]	+	+	+ [61]
*Msx2*	-	+	+ [61]	-	-	- [61]	+	-	+ [61]	-	-	+ [61]
*Msx3*	-	-	n/a	-	+	n/a	+	+	n/a	-	+	n/a
*Pitx2*	-	+	- [51]	-	+	- [51]	-	+	- [51]	-	+	- [51]

Summary of gene expression patterns in the mesenchymal compartment of the catshark scale and tooth buds, in comparison to data published for molar bud development in the mouse. Positive expression is represented by a +, negative expression by a –, and references for expression in mouse molar buds are cited in the table, except when non available (n/a)

**Table 2:** Summary of epithelial gene expression patterns

Stage	EM			LM			ED			LD		
	Scale	Tooth	Molar	Scale	Tooth	Molar	Scale	Tooth	Molar	Scale	Tooth	Molar
*Msx1*	-	+, +oe	- [61]	-	+, +oe	- [61]	-	+	- [61]	+	+cusp	- [61]
*Msx2*	-	+, +oe	- [61]	+	+	+ek [61]	+	+ lab	+, +oe [61]	+	+cusp	+oe [61]
*Msx3*	-	+	n/a	+	+	n/a	+	+ lab	n/a	+	+cusp	n/a
*Pitx1*	+	+, +oe	+ [52]	+	+, +oe	+ [52]	+root, +oe	+, +oe	+, +oe [52]	-, +oe	+, +oe	+ [52]
*Pitx2*	-	+, +oe	+ [15]	-	+, +oe	+ [15]	-	-, +oe	+, +oe [52]	-	-, +oe	+, +oe [52]
*Shh*	-	+tip	+ [62]	+	+tip	+ek [62]	+	+ tip	+ek [62]	+	+cusp	+ [63]
*Fgf8*	-	-	+ [48]	+top	+tip	- [48]	+top	+ tip	- [48]	-	+cusp	- [48]
*Bmp4*	-	+	-	-	+lab, +oe	+ek	+top	+	+ek	+top	+lab cusp	+ek
		+oe	[46]			[46]			[46]			[46]

Summary of gene expression patterns in the epithelial compartment of the catshark scale and tooth buds, in comparison to data published for molar bud development in the mouse. Positive expression in dental epithelium is represented by a +, negative expression by a –, and references for expression in mouse molar buds are cited in the table, except when non available, n/a. Further detail is given when necessary: regionalized expression within the dental epithelium: enamel knot (ek), tip of the tooth bud (tip), top of the scale bud (top), restriction on the lingual (ling) or labial (lab) side of the tooth, cuspid iterative expression (cusp), root of the scale (root) or outer dental epithelium (oe)

### Divergent dynamics of expression patterns in the mesenchyme

*Msx* genes are mesenchymal markers expressed before stage EM in the mouse molar. They are also transcribed before morphogenesis in the catshark odontogenic band (Fig. 2) but the mesenchymal expression of *Msx1* is down-regulated at the beginning of tooth morphogenesis and then up-regulated later in the catshark (LM in scales and ED in teeth). This transcription factor is then expressed in the mesenchyme of all structures until stage LD. Msx1 was shown to regulate *Bmp4* expression in mouse tooth bud mesenchyme but not in the epithelium [11, 12]. In the catshark, we show that this situation may also happen in scale buds but not in tooth buds that are devoid of mesenchymal *Bmp4* expression. A continuous expression of *Msx* members is maintained in the mesenchyme of catshark scales and teeth starting at LM stage. A more precise comparison with mouse tooth buds, including the different members of the gene family, is still complicated as no expression data is published for mouse *Msx3* in tooth development, and because of redundancy in Msx1 and Msx2 function in mouse tooth bud mesenchyme [45]. Overall, our results suggest that the early mesenchymal Msx1-Bmp4 relationships described in mouse molar development are not functional in shark tooth development. However, they may involve other *Bmp* gene family members as *Bmp4* but also other members of the Bmp family have been proposed as a major marker of the transition of competency from the epithelial compartment to the mesenchymal compartment in mouse tooth bud morphogenesis [46]. Our observation in the catshark would suggest that this transition of competency might happen through the expression of *Bmp4* only during scale bud development.

### Relative expression domains of Bmp, Shh and Fgf in the epithelium

*Bmp4* expression pattern in the epithelium of mouse molar development is very well documented in particular in the very restricted zone of the enamel knot within the inner dental epithelium [46]. At bell stage but before ameloblast differentiation, *Bmp4* is expressed in the putative secondary enamel knots [46]. Expression of *Bmp4* in shark scales and teeth also displayed restricted zones of expression patterns. In scale buds, expression started at ED and was then restricted to the top of the scale bud. In tooth buds, expression started at LM and was restricted to its labial side at the LD stage. The shape differences between scale and tooth buds do not allow easy comparisons of expression patterns but insights can be gained thanks to *Shh* and *Fgf8* expression.

Both *Shh* and *Fgf8* displayed a focal distribution of their mRNAs in epithelial cells during tooth and scale bud development in the catshark. Their first expression could be detected at stage EM and LM respectively, and were located at the distal tip of the developing tooth bud and consequently in the forming secondary cusp of teeth at LD stage (Fig. 4). The *Fgf8* zone of expression appeared always included in, but smaller than, the zone of *Shh* expression, which is very comparable to the respective expression patterns of *Shh* and *Fgf4* in mouse molar enamel knots [47]. Note that in mouse, *Fgf8* is not one of the *Fgf* genes which show restricted expression in the enamel knot: *Fgf4* and *Fgf9* [48], as well as *Fgf15* and *Fgf20* [49] display such an expression pattern while other *Fgfs* have very different expression patterns. In the catshark, we could not describe the expression patterns of the specific *Fgf* genes involved in the enamel knot activity in the mouse because of the lack of sequence data. However, we show that the FGF signaling pathway is expressed in a relatively small part of the catshark dental epithelium. A similarly nested expression pattern of *Shh* and *Fgf8* was observed in developing scale buds, with *Shh* expressed in the planar distal zone of the bud epithelium, and *Fgf8* in the center of this zone. The observed restricted expression patterns of these signaling molecules and transcription factors call for a potential enamel knot-like system during tooth and scale morphogenesis in the catshark.

### Shark teeth: cusps without an enamel knot

**Fig. 8:**
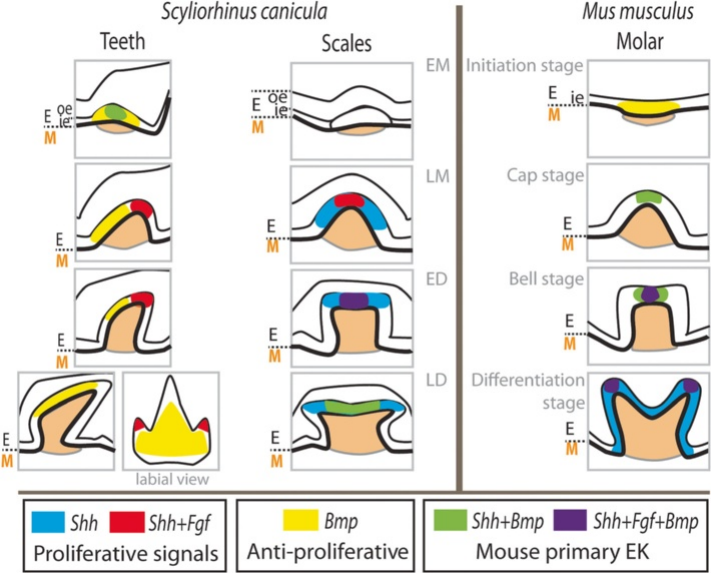
A summary of epithelial gene expression patterns in tooth and scale buds of the catshark *Scyliorhinus canicula*, in comparison to mouse molar development. Co-expression of anti- and pro-proliferative signals are colored similarly in shark tooth and scale buds and mouse tooth buds, *Fgf* and *Bmp* zones of expression correspond to *Fgf8* and *Bmp4* in the catshark, *Bmp4* and *Fgf4* in mouse (following [17]). E: epithelium; M: mesenchyme; oe: outer epithelium; ie: inner epithelium

In mouse, the molar primary enamel knot is defined both at the cytological and gene expression levels: characteristics include a non-proliferative, tightly packed group of cells with secretion of both pro-proliferation (Shh, Fgf) and pro-differentiation (Bmp) signals, with a finally apoptotic fate [9]. We first checked the cytological characteristics of tooth epithelial cells during the catshark tooth morphogenesis: the folded tip of the tooth bud does not show any histological specificity compared to the surrounding epithelium but is made of much more slowly proliferating cells (see Fig. 6). In terms of gene expression, this tip of the bud is the specific site of co-expression of *ScShh* and *ScFgf8* (see Fig. 8). However, the expression of *ScBmp4* was not co-localized with the *Shh* and *Fgf* center of expression, as it was found on the labial side of the tooth bud epithelium, which also was the zone of stronger proliferation. The Bmp signal in mouse molar enamel knot is also characterized by the expression of *Bmp2* and *Bmp7*, so further description of expression patterns for other members of the Bmp gene family might be helpful in the future to fully compare shark and mouse tooth development, and support our hypothesis that a second signaling center occurs in the labial epithelium of shark tooth buds.

From our results, it appears that mouse molar development is more comparable to scale than to tooth development in the catshark (Fig. 8). Co-expression of *ScFgf8* and *ScBmp4* is found in a narrow focal part of the apical epithelium of the scale bud displaying low to no proliferation, while *ScShh* shows a larger but still apically restricted zone of expression. This observation is similar to the *Shh* expression in a larger part of the enamel knot than for *Bmp4* and *Fgf4* in mouse [17]. The signaling center described in the scale bud is however not strictly equivalent to what has been described in the primary enamel knots of the mouse molar because of the lack of apoptosis.

### Outer epithelium patterning

*Pitx* gene expression patterns are very similar in scales and teeth: *Pitx3* was not expressed in shark tooth or scale (and is not expressed in mouse molar) while *Pitx1* is faintly expressed at the beginning of tooth and scale development in the bud dental epithelium until the LD stage. Additional *Pitx2* faint expression is detected over shark tooth development, as also described during mouse molar development, but expression of this gene was not detected during scale development. Expression of *Pitx* genes shows the peculiarity of asymmetry in the outer dental epithelium: in shark tooth buds, *ScPitx1* and *ScPitx2* are expressed in the lingual side of the EM and LM buds. On the other hand, the expression of *ScPitx1* is found as a complete circle surrounding the root of the scale bud, as well as in a distal spot directly covering the scale bud top, therefore displaying no asymmetry in scale buds (see Figs. 3, 4 and 5). *Pitx1* and/or *Pitx2* expression in the mouse and teleost fish was also reported in the outer dental epithelium without any specific asymmetry in their expression patterns [15, 50–52]. Our results suggest that the outer dental epithelium of scale and tooth buds in the catshark is patterned.

### Enamel knots and growth direction

We speculate that the discrepancy in the location of signals involved in shark tooth and scale morphogenesis is associated with the growth direction of the final structures. As shown in Fig. 1, teeth in shark display growth axis bent towards the labial side of the jaw associated to a final overall bilateral symmetry. On the other hand, primary scales are round, with a radial organization following their growth along an apical-basal axis. This growth axis is detectable through the expression patterns of *Pitx* genes that are found in the epithelium surrounding the growing bud (herein called outer epithelium): expression of *ScPitx1* delineates the basal circle within which the scale bud grows, as well as the top of the growing bud. This expression is therefore following a radial symmetry. In contrast, *Pitx1* and *Pitx2* expression is asymmetric around the tooth bud marking the lingual side of the tooth bud at LM and ED, therefore pointing the growth direction of the bud. In mouse, *Pitx1* is transcribed in all layers of the dental epithelium, except in the enamel knot making its expression radially symmetrical as in scale bud development [15, 52]. From these results, we suggest that two mechanisms may account for the growth topology in shark teeth and scales: (i) heterogeneity in Pitx transcription factor expression in the outer dental epithelium and (ii) physical exclusion or superposition of Shh/Fgf and Bmp signaling pathways in the dental epithelium. It is important to note that later developing scales in the catshark and most species of sharks display a great variability in their shape, going from rounded, single axis, structures (such as the scales described in this work) to elongated and bent, teeth-like, structures [53]. Variations in the relative strength of these signals may therefore be responsible for the variations observed in the morphology of dermal scales. Further work including other types of dermal scales is therefore needed to test this hypothesis and the similarity between tooth and bent dermal scale morphogenesis.

### Evolutionary perspectives

**Fig. 9:**
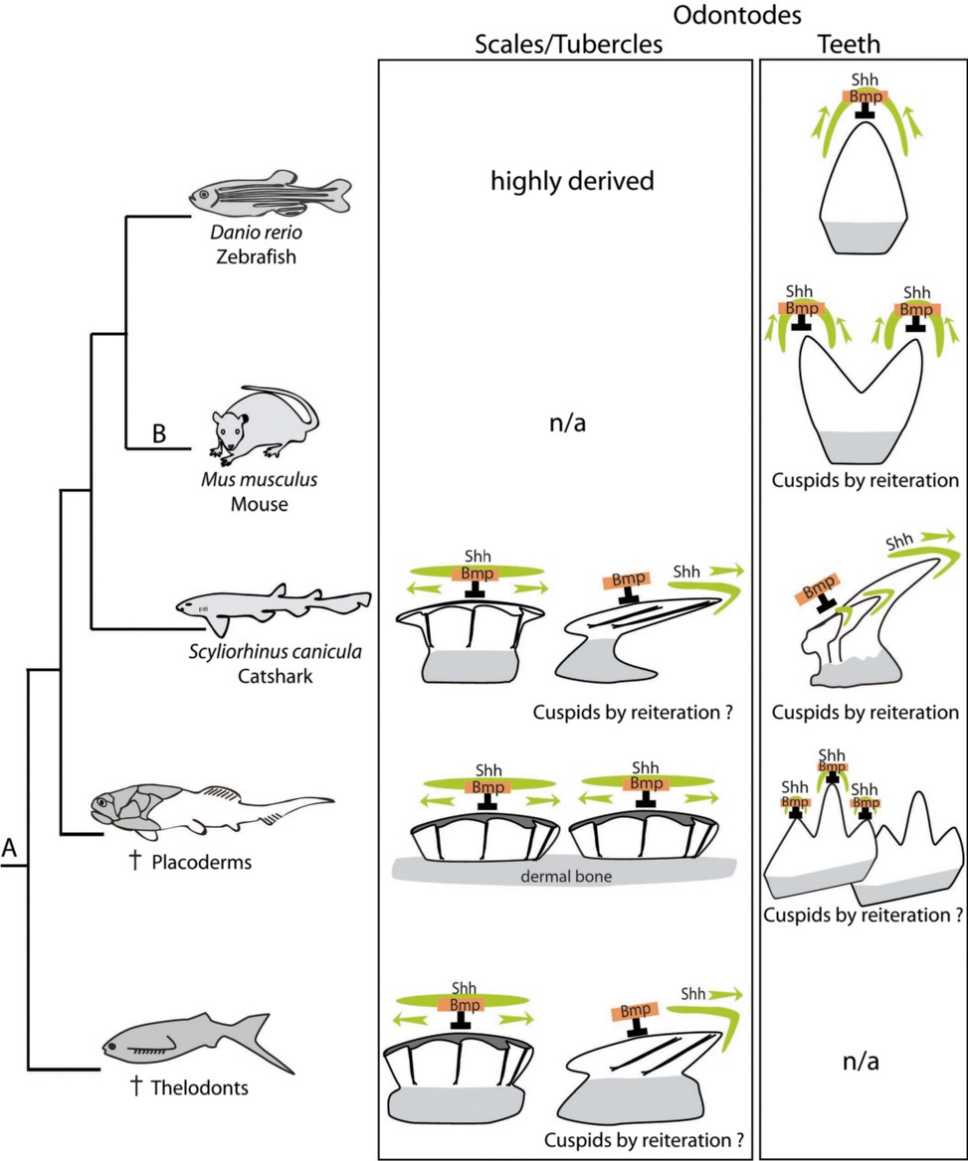
A model of evolution for the regulatory system involved in odontode morphogenesis in the course of vertebrate diversification. The phylogenetic framework is from [56] and the odontode structures (lateral views) are from [55]. The relative expression domains of *Shh* and *Bmp*s are indicated in green and orange respectively. The orientation of growth, following the putative Shh signal, is indicated by a green arrow. Odontodes are an ancestral character for vertebrates that first occurred as outer mineralized structures (*A*). In particular, thelodonts display both radially symmetrical and bilaterally symmetrical scales. A strict coupling of the Shh and Bmp pathways involved in cusp growth is currently described only in mammals (*B*) but may be proposed as a mechanism for cuspid growth in placoderms

Teeth and scales in vertebrates were described as odontodes: repetitive structures found in different sites of the body, made of similar tissues, and developing through similar mechanisms [54]. It has been previously shown that early stages of tooth and scale development in the catshark displayed similar developmental gene expression patterns [4], while this study shows different location of the signaling centers involved in cell proliferation as well as differential patterning of the surrounding epithelium. These two situations also differ from the growth mechanisms found in the mouse molar with the activity of a very specific signaling center: the enamel knot [17]. To trace back the evolution of these three modes of odontode growth, the fossil record is of great interest as most of the remains from early vertebrates and gnathostomes actually are teeth and denticles, associated or not to dermal bones (Fig. 9 and [55]). Data from the paraphyletic assemblage of placoderms (today considered sister-groups to extant gnathostomes [56]) show that tooth-like structures (both on the jaw margin and pharyngeal elements) and dentineous dermal bone tubercles have a single growth axis [34, 57, 58]. Letting aside the mineralized structures of the conodonts, for which much debate is still on-going about their relationship to vertebrates, the earliest remains that can be described as odontodes outside of gnathostomes are the single dermal denticles found in thelodonts [34], where scales can be both button-like (similar to the catshark scales displayed here) or elongated and posteriorly bended like shark teeth (e.g. [53 and 59]). Therefore, both types of odontode growth described in extant chondrichthyes seem to be already present in one of the earliest vertebrates. Cuspidal structures are observed in virtually all fossil and extant groups: shark scales can be tricuspidate in some places of their body, placoderm “tubercles” on jaw-bones and body dermal bones are multi-cuspidate [58], teleost fishes display a variety of tooth shapes including multi-cuspidate ones [33], and mammals have variations in the number of cuspids on their teeth [60]. In this context, we propose the hypothesis of an vertebrate ancestral morphogenesis regulation system with independency between the pro-proliferation and anti-proliferation signals (as in thelodonts and sharks) explaining the ability to grow single-axis and bent-axis structures, and a later evolutionary event leading to the enamel knot system in the lineage leading to extant mammals (Fig. 9).

## Conclusions

This work intended to test the hypothesis of homologous regulatory systems at work both in the catshark and the mouse tooth morphogenesis. This hypothesis was based on the well-described role of an enamel knot in the mouse developing molar, involved in the regulated growth of cusps. Our results showed that homologous regulatory pathways are expressed during tooth morphogenesis in both species (Shh, Bmp and Fgf) but that no cytological or gene expression data support the hypothesis of a primary enamel knot in sharks. In particular, a separation between pro-proliferation (Shh and Fgf) and pro-differentiation (Bmp) signals support the existence of two separated signaling centers.

We also compared expression of these regulatory pathways in tooth and scale of the catshark and detected a series of variations which strongly contrast with previous work on the expression of early actors of scale and tooth morphogenesis [4]. One striking result from our work is a stronger similarity of gene expression patterns between mouse tooth and catshark scale development. We suggest that these results are linked to the direction of bud growth, which is along an apical-basal axis in mouse molars and shark scales, while shark teeth grow in a bent labial-lingual axis. These results open new horizons to diversify our models of tooth growth as proposed by Salazar-Ciudad and Jernvall [24] and further understand the developmental origins of tooth morphological evolution.

## Availability of supporting data

All the supporting data are included as additional files.

## Additional files

Additional file 1:
**Neighbor joining phylogenetic reconstructions of gene relationships in jawed vertebrates.** BMP, Fgf and Msx protein sequences from various species were extracted from the NCBI database (NCBI accession numbers are indicated on each leaf of the tree) and were aligned with the translated sequences of the catshark *Scyliorhinus canicula* cDNA sequences to be identified, using ClustalX [64] with manual optimization using MUST software [65]. Regions of ambiguous homology were removed. Evolutionary distances (using JTT + gamma = 0.6 model) were computed and a neighbor-joining tree was obtained using MEGA6 [66]. The robustness of the tree nodes was estimated by a bootstrap test (200 replicates). **A**: phylogenetic tree (total 361 sites) including the Bmp2 and Bmp4 paralogy groups, identification of the catshark Sc*Bmp4* sequence; **B**: phylogenetic tree (total 144 sites) including the Fgf8, Fgf17 and Fgf18 paralogy groups, identification of the catshark Sc*Fgf8* sequence; **C**: phylogenetic tree (total 135 sites) including the Msx1, Msx2 and Msx3 paralogy groups, identification of the Sc*Msx1*, Sc*Msx2* and Sc*Msx3* sequences; **D**: phylogenetic tree (total 200 sites) including the Pitx1, Pitx2 and Pitx3 paralogy groups, identification of the Sc*Pitx1*, Sc*Pitx2* and Sc*Pitx3* sequences. (PDF 104 kb) Additional file 2:
**Apoptosis detection in developing tooth buds in the catshark** ***Scyliorhinus canicula.*** Animation of longitudinal successive merged views (from surface to internal) by confocal z-imaging of a whole-mount jaw after staining of apoptotic nuclei (TUNEL, red), and counter-staining against actin (phalloidin, red) and DNA (DAPI, blue). Please note the scale on the merged image is not correct. (AVI 25347 kb) Additional file 3:
**Apoptosis detection in developing scale buds in the catshark** ***Scyliorhinus canicula.*** Animation of successive merged views by confocal z-imaging of a of a thick transversal slice of a tail: apoptotic nuclei (TUNEL, red), and counter-staining against actin (phalloidin, red) and DNA (DAPI, blue). (AVI 23043 kb)
