# Supplementary figures and images for: Tooth and scale morphogenesis in shark: an alternative process to the mammalian enamel knot system
Source: BMC Evol Biol. 2015 Dec 24;15:292. doi: 10.1186/s12862-015-0557-0 (PMC4690397; doi:10.1186/s12862-015-0557-0)

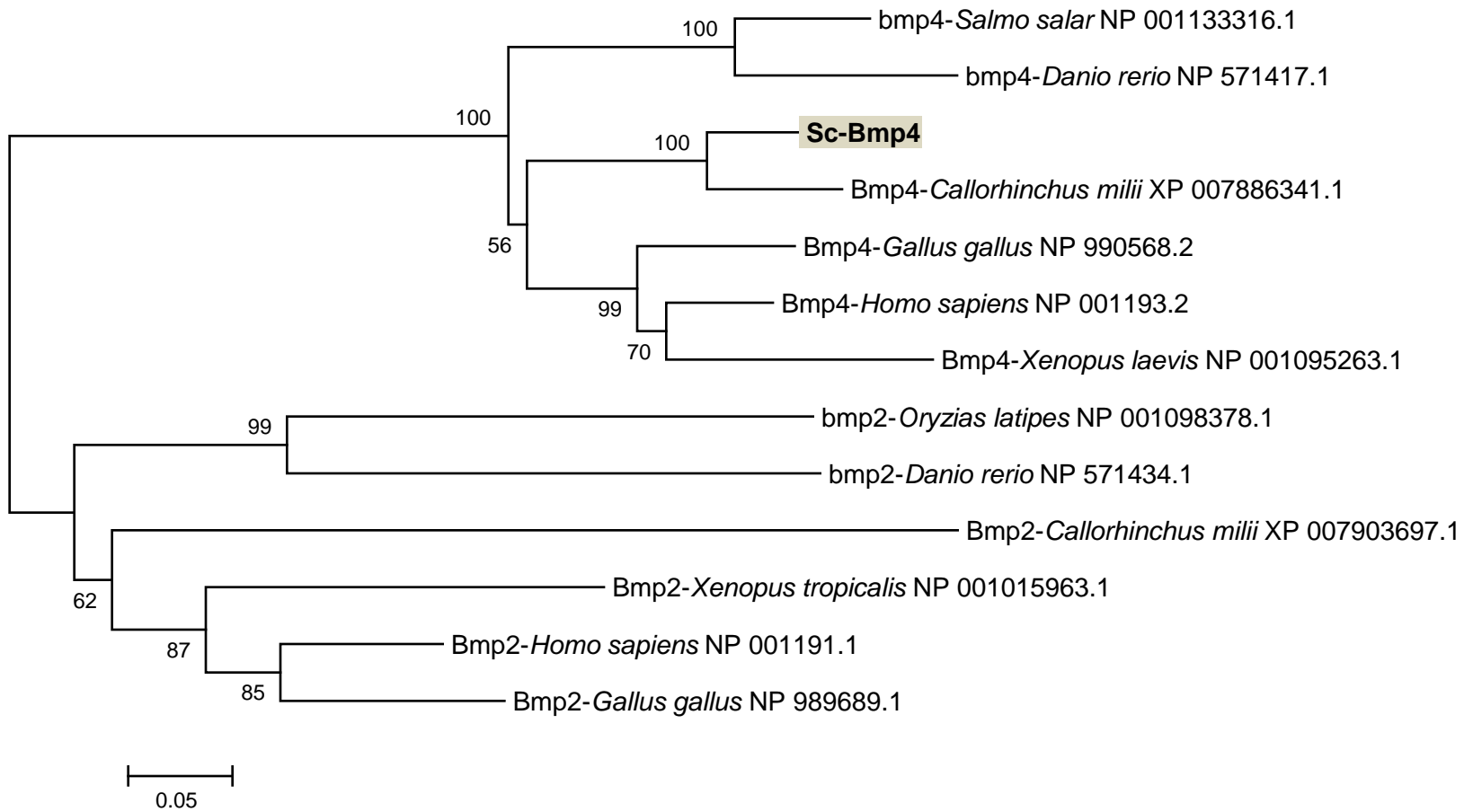

A.

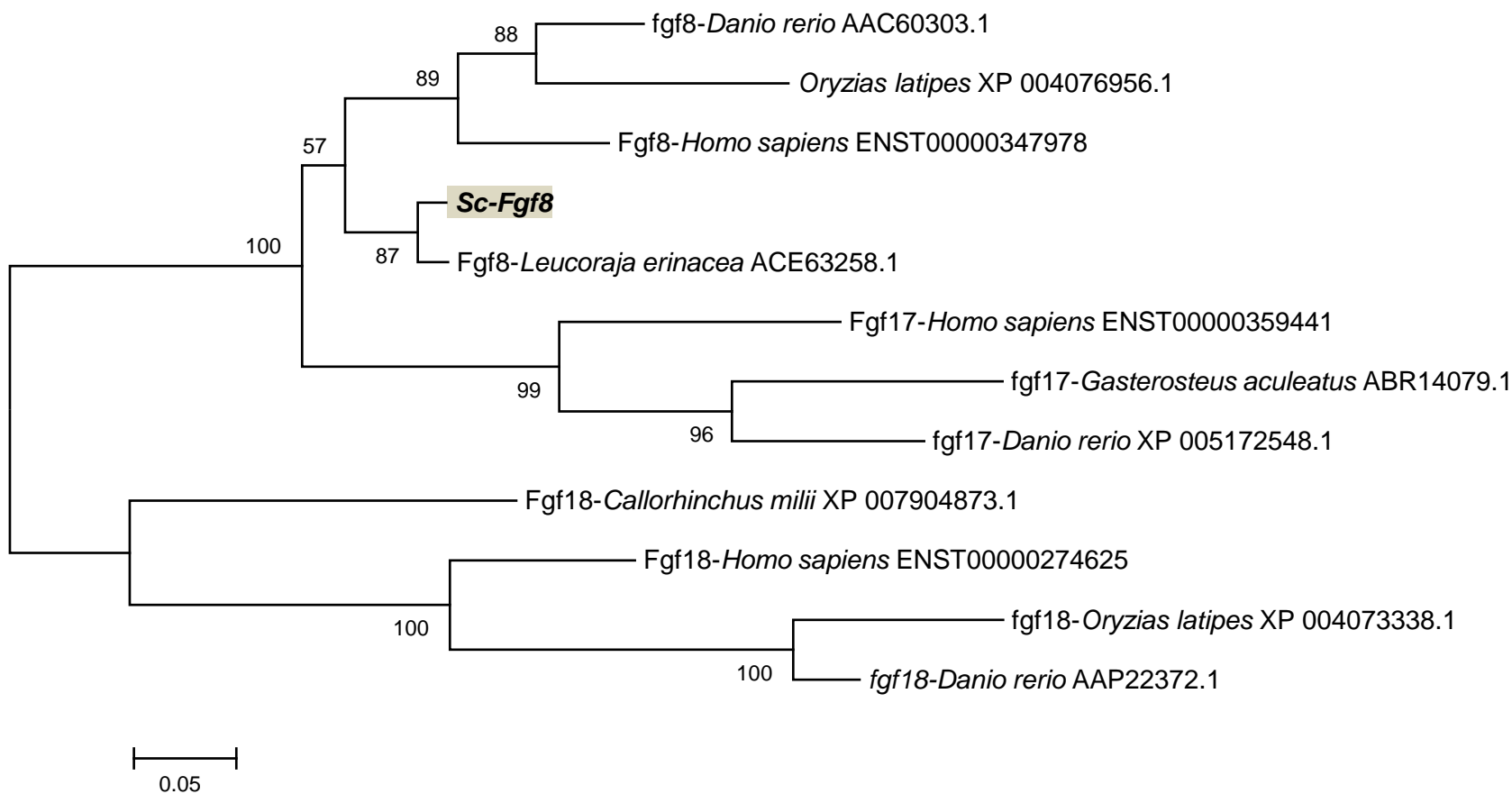

B.

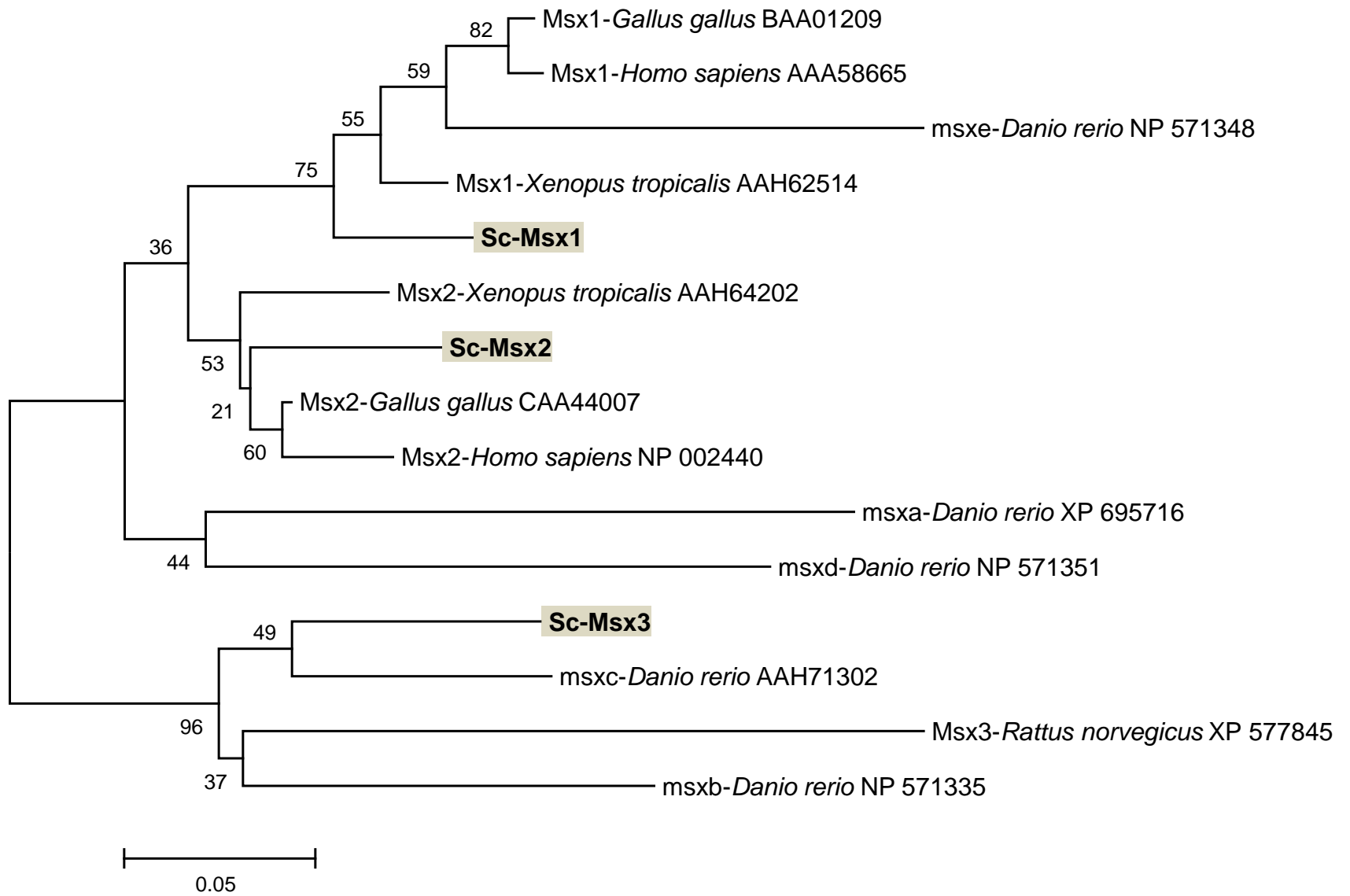

C.

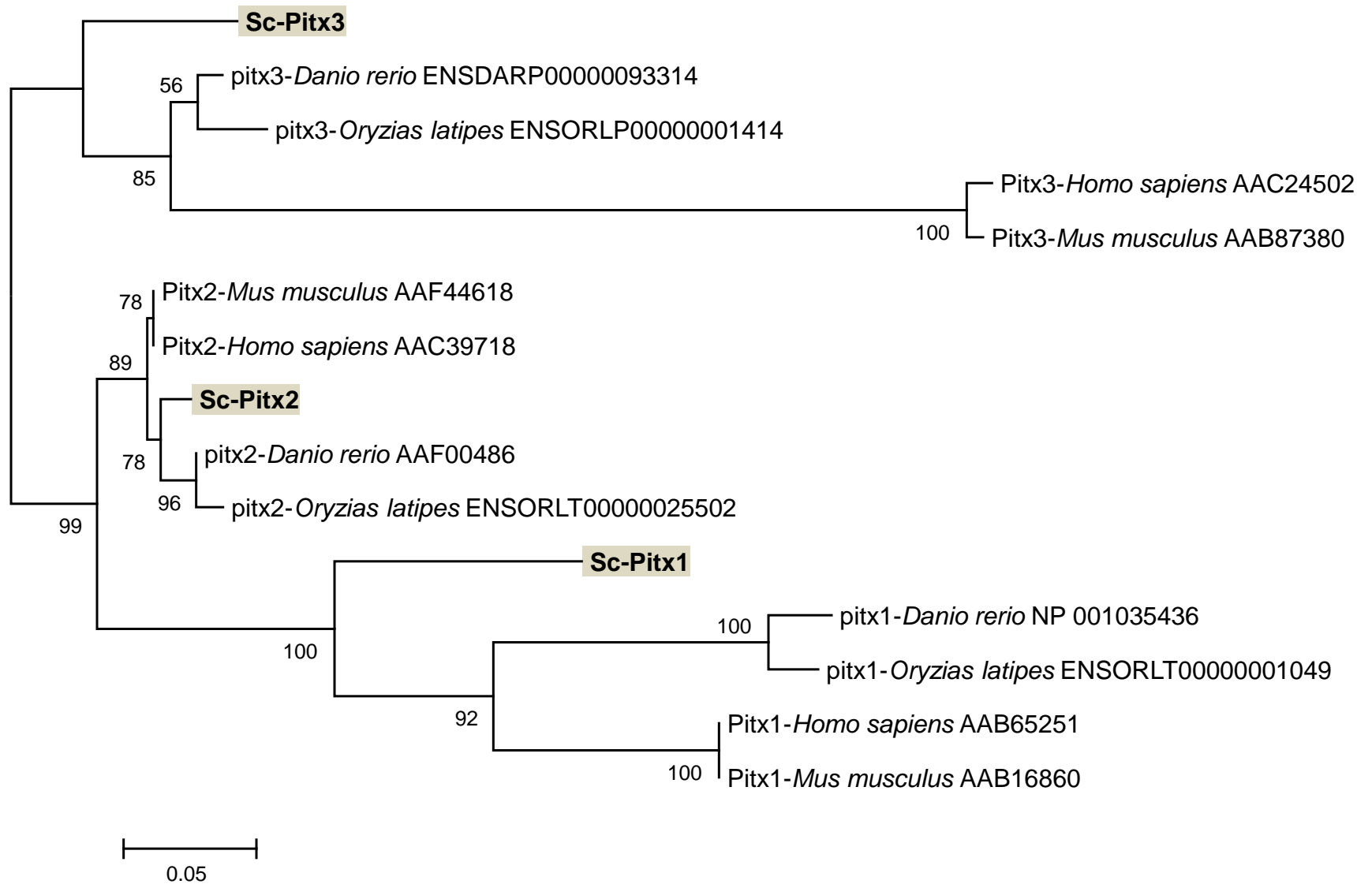

D.

Supplement: Additional file 1: — Neighbor joining phylogenetic reconstructions of gene relationships in jawed vertebrates. BMP, Fgf and Msx protein sequences from various species were extracted from the NCBI database (NCBI accession numbers are indicated on each leaf of the tree) and were aligned with the translated sequences of the catshark Scyliorhinus canicula cDNA sequences to be identified, using ClustalX [64] with manual optimization using MUST software [65]. Regions of ambiguous homology were removed. Evolutionary distances (using JTT + gamma = 0.6 model) were computed and a neighbor-joining tree was obtained using MEGA6 [66]. The robustness of the tree nodes was estimated by a bootstrap test (200 replicates). A: phylogenetic tree (total 361 sites) including the Bmp2 and Bmp4 paralogy groups, identification of the catshark ScBmp4 sequence; B: phylogenetic tree (total 144 sites) including the Fgf8, Fgf17 and Fgf18 paralogy groups, identification of the catshark ScFgf8 sequence; C: phylogenetic tree (total 135 sites) including the Msx1, Msx2 and Msx3 paralogy groups, identification of the ScMsx1, ScMsx2 and ScMsx3 sequences; D: phylogenetic tree (total 200 sites) including the Pitx1, Pitx2 and Pitx3 paralogy groups, identification of the ScPitx1, ScPitx2 and ScPitx3 sequences. (PDF 104 kb) [file 12862_2015_557_MOESM1_ESM.pdf]
